# Risk of Recurrent Venous Thromboembolism in Selected Subgroups of Men: A Danish Nationwide Cohort Study

**DOI:** 10.1055/a-1949-9404

**Published:** 2022-11-18

**Authors:** Ida Ehlers Albertsen, Stavros V. Konstantinides, Gregory Piazza, Samuel Z. Goldhaber, Torben Bjerregaard Larsen, Mette Søgaard, Peter Brønnum Nielsen

**Affiliations:** 1 Department of Cardiology, Aalborg University Hospital, Aalborg, Denmark; 2 Aalborg Thrombosis Research Unit, Aalborg University, Aalborg, Denmark; 3 Center for Thrombosis and Hemostasis, Johannes Gutenberg University, Mainz, Germany; 4 Department of Cardiology, Democritus University of Thrace, Greece; 5 Division of Cardiovascular Medicine, Brigham and Women's Hospital, Harvard Medical School, Boston, Massachusetts, United States

**Keywords:** venous thromboembolism, recurrence, sex, men, anticoagulation, treatment

## Abstract

**Background** Although men are considered at high risk for recurrent venous thromboembolism (VTE), sex-specific data on prognostic factors are lacking. We estimated the cumulative recurrence risks associated with clinical characteristics and comorbidities known or suspected to be associated with the development of VTE recurrence: major surgery, trauma, history of cancer, rheumatic disorder, ischemic heart disease, congestive heart failure, chronic obstructive pulmonary disease, diabetes, chronic renal disease, varicose veins, alcohol-related diseases, and arterial hypertension.

**Methods** We linked nationwide Danish health registries to identify all incident VTE in- and outpatients in men from 2008 through 2018. Recurrent VTE risk 2 years after anticoagulant discontinuation was calculated using the Aalen-Johansen estimator, stratified by age above/below 50 years.

**Results** The study included 13,932 men with VTE, of whom 21% ( *n* = 2,898) were aged <50 years. For men aged <50 years with at least one of the clinical characteristics, 2-year recurrence risk ranged from 6% (major surgery) to 16% (history of cancer). For men ≥50 years with at least one of the characteristics, recurrence risk ranged from 7% (major surgery) to 12% (ischemic heart disease, chronic obstructive pulmonary disease, and chronic renal disease). Men aged <50 and ≥50 years without the clinical characteristics all had a recurrence risk of 10%.

**Discussion** We demonstrated a 2-year recurrence risk of at least 6%, regardless of age category and disease status, in this nationwide cohort of men with VTE. The recurrence risk must be balanced against bleeding risk. However, the high recurrence risk across all subgroups might ultimately lead to greater emphasis on male sex in future guidelines focusing on optimized secondary VTE prevention.

## Introduction

Venous thromboembolism (VTE), encompassing both deep vein thrombosis (DVT) and pulmonary embolism (PE), is the third leading vascular disease after myocardial infarction and stroke. 1 Patients with incident VTE carry a considerable risk of recurrence with a high risk of morbidity and mortality. 2 3 Within 10 years after a first VTE, more than 20% will experience recurrence, regardless of the etiology of the first event. 4 Accordingly, anticoagulation constitutes the foundation of VTE treatment and prevention of recurrence for a majority of patients.

Assessment of VTE recurrence risk after acute VTE is complex. Management with long-term anticoagulation must be weighed against the long-term risk of major bleeding associated with anticoagulant therapy. Previous studies have reported that men have an overall higher VTE recurrence risk compared with women. 5 6 In a meta-analysis from 2011, men had a 2.2-fold higher risk of recurrence compared with women. 6 In a 2019 a meta-analysis on patients with “unprovoked” (no identifiable risk factors for VTE) VTE, the 10-year cumulative risk for men was 41% (95% confidence interval 28–56%) and 29% (95% confidence interval 20–38%) for women, respectively. 7 According to contemporary European guidelines, patients should be considered candidates for extended anticoagulant treatment if their estimated risk for long-term recurrence exceeds 3% per year. 8 However, VTE patients may have heterogeneous risk profiles with various underlying chronic conditions, risk factors for major bleeding, and recurrence rates. 4 9 10 We therefore hypothesized that treatment decisions may benefit from a more differentiated and personalized strategy. Specifically, we estimated crude cumulative recurrence risks associated with clinical characteristics and comorbidities (recent major surgery, recent trauma, history of cancer, rheumatic disorder, recent ischemic heart disease, congestive heart failure, chronic obstructive pulmonary disease, diabetes, chronic renal disease, varicose veins, alcohol-related diseases, and arterial hypertension) known or suspected to be associated with an increased risk of developing VTE recurrence.

## Patients and Methods

### Study Design

This study was a retrospective observational cohort study based on secondary data collection from Danish nationwide administrative databases, with a study period from 2008 through 2018.

### Data Sources

This study was based on linkage of three nationwide Danish registries: (1) The Danish National Patient Registry which has tracked hospitalizations since 1977, and outpatient and emergency department visits since 1995 11 ; (2) The National Prescription Register, which holds detailed information on purchase date, Anatomical Therapeutic Chemical (ATC) classification code, package size, and dosage for every prescription dispensed in Denmark since 1994 11 ; and (3) The Danish Person Registry, which contain data on gender, date of birth, vital and emigration status. 12 All codes used in this study are presented in Supplementary eTable 2 .

### Study Population

The source population covered all residents of Denmark from 2008 through 2018. The study population comprised all men with a first inpatient or outpatient diagnosis of VTE in the National Patient Register. Both primary and secondary VTE diagnoses were considered for inclusion. Of note, the primary diagnosis describes the diagnosis that was the most serious and/or resource-intensive during the hospitalization or the inpatient encounter, whereas secondary diagnoses are used for conditions that coexist at the time of admission, or develop subsequently, and that affect the patient care for the current episode of care. Emergency department diagnoses were excluded because of a low positive predictive value of 31% in the Danish registries. 13 The date of the incident VTE diagnosis was defined as the index date for baseline assessments.

To ensure enough clinical record history for risk factors, we excluded patients who had not been residents in Denmark for at least 5 years prior to VTE diagnosis. Patients were required to be age 18 years or older at the time of incident VTE. They were considered for inclusion if they redeemed a prescription for oral anticoagulation within 30 days after the incident VTE, ensuring a positive predictive value of 90% for the VTE diagnosis. 14 The duration of the anticoagulant treatment period was defined using information on package size and prescription frequency in the Danish Prescription Registry, 15 including a grace period to allow gaps of maximum 30 days. 16 Since we were interested in estimating recurrence risk without anticoagulation, patients with a treatment period lasting more than 1 year were excluded. We also excluded patients with a diagnosis of atrial fibrillation or heart valve replacement at baseline or after the VTE event in view of the life-long indication for anticoagulation. Finally, we excluded patients with cancer diagnosis in the year prior to the incident VTE since these patients constitute a special VTE population in terms of recurrence risk and need for long-term antithrombotic treatment. 17

### Patient Characteristics

The investigated characteristics and comorbidities were based on risk factors described by the International Society on Thrombosis and Haemostasis (ISTH) in combination with relevant guidelines and previous literature. 4 8 18 19 The recurrence risk was investigated according to the following characteristics: recent major surgery, recent trauma, history of cancer, rheumatic disorder, recent ischemic heart disease, congestive heart failure, chronic obstructive pulmonary disease, diabetes, chronic renal disease, varicose veins, alcohol-related diseases, and arterial hypertension. Patient characteristics were identified either dating 3 months or 10 years prior to the incident VTE dependent on the persistent or transient nature of the disease. Comorbidity status and patient characteristics were established by coded hospital records in the registries. Co-medication was based on prescription data within 1 year prior to inclusion. Bleeding during anticoagulant treatment was defined as patients with a hospital diagnose code of gastrointestinal bleeding or intracranial bleeding coded during their anticoagulant treatment period.

Additionally, to describe our cohort, we used the “AIM-SHA-RP” risk score (age, incident PE, major surgery, statin treatment, heart disease, antiplatelet treatment, chronic renal disease, and Pneumonia/sepsis [ Supplementary eTable 1 ].) to categorize patients as having an estimated low (<5%), intermediate (5–10%), or high (>10%) 2-year recurrence risk after anticoagulation discontinuation. 20

### Outcome

Patients were followed in the registries for 2 years after anticoagulation was discontinued for the occurrence of recurrent VTE. To ensure the validity of the outcome, hospital discharge diagnosis of recurrence was required to be the primary in-hospital or ambulatory diagnosis with a confirmatory imaging examination. According to a validation study, this approach for identifying recurrent VTE ensured a positive predictive value of 82%. 21

### Statistical Analysis

Patient characteristics at the time of incident VTE were described using proportions for categorical variables and means with standard deviation (SD) for continuous variables. Since VTE risk rises steeply after the age of 50 years and because age >50 years previously has been associated with a high recurrence risk among men, we stratified all analyses by age 50 years or less. 20 22 23

Follow-up began at the date of discontinuation of anticoagulation. We used the Aalen-Johansen estimator, assuming death as competing risk, to estimate the cumulative risk of recurrence at 2 years after discontinuation of anticoagulation for patients with and without major chronic diseases and relevant characteristics, respectively. End of study (January 2019), death, re-initiation of anticoagulation, and emigration were considered as censoring events.

For comparison with other studies, incidence rates for recurrence were calculated as the number of events per 100 person-years. The associated risk was not compared among those with or without the clinical characteristic, thus confounding was not a concern in our descriptive approach of estimating the risks.

### Supplementary and Sensitivity Analyses

Since DVT and PE present as different diseases with variable prognoses, 3 we repeated the main analyses with restriction to patients with DVT versus PE, to assess whether recurrence risk differed by the type of VTE. If a patient had a diagnosis of both PE and DVT, preference was given to the PE diagnosis. Additionally, a sensitivity analysis was done allowing for 18 months initial standard therapy instead of 1 year. Also, a sensitivity analysis was done repeating the main analysis with age stratified according to the median and 75 percentile (not including 25 percentile because this cut was identical to the main analysis). Finally, for clarifying long-term risk of recurrence, we also reported results with 5-years follow-up.

Analyses were performed using Stata/MP version 15 (StataCorp LP). This study was conducted in compliance with the General Data Protection Regulation and is part of North Denmark Region's record of processing activities (j.no. 2017-68). Institutional Review Board approval is not required for registry-based studies in Denmark.

## Results

**Fig. 1:**
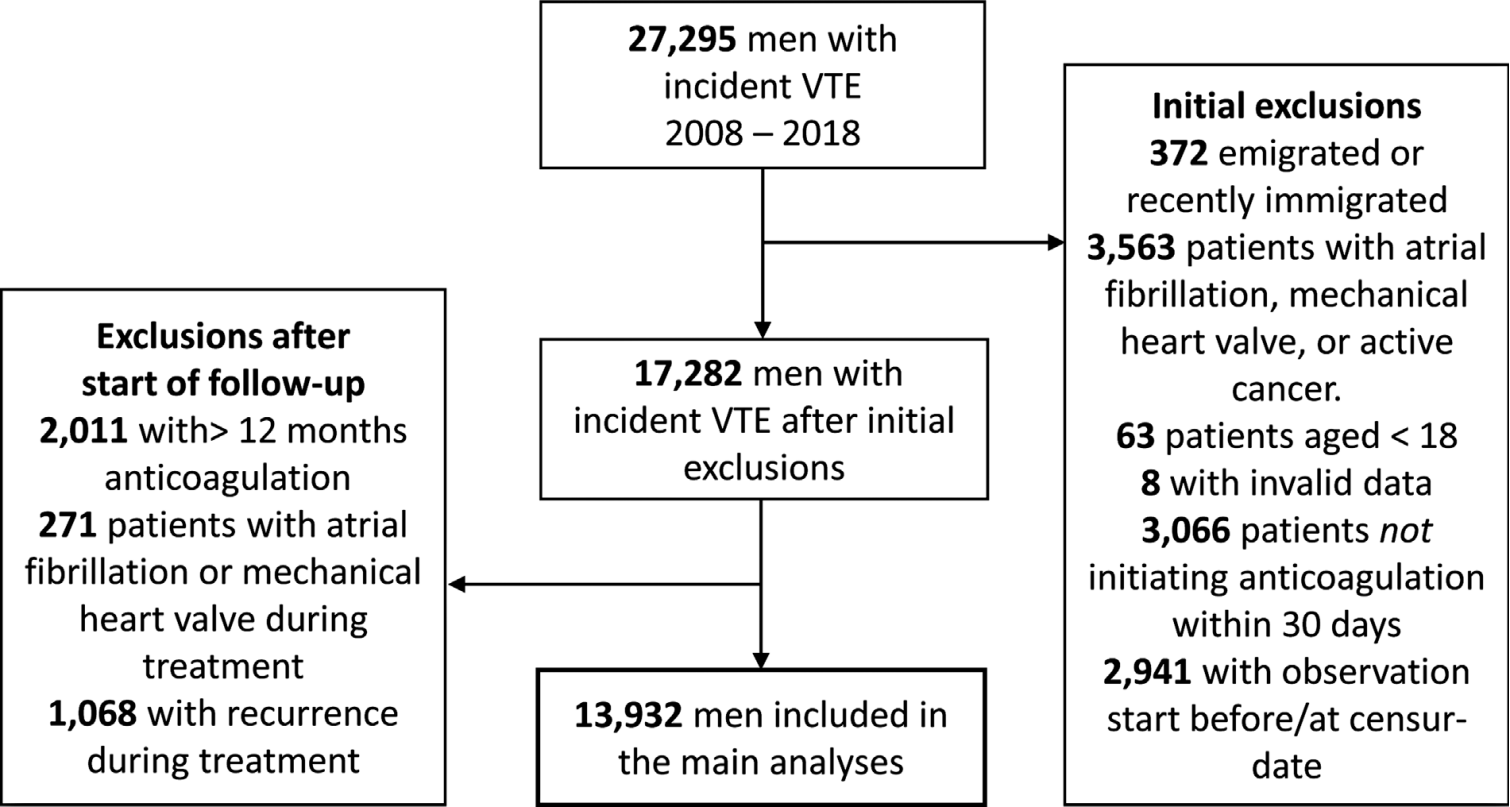
Flowchart of the study population.

The study population comprised 13,932 men with incident VTE from 2008 through 2018 ( Fig. 1 ). Of these, 21% ( *n* = 2,898) were aged <50 years (mean age 40.3 [SD 7.8]) and 79% ( *n* = 11,034) were aged ≥50 years (mean age 67.7 [SD 10.3]). Baseline characteristics are presented in Table 1 . Among men aged <50 years, 27% presented with PE, whereas 37% of men aged ≥50 years had PE. Most men were categorized as having a high (>10%) 2-year recurrence risk (AIM-SHA-RP score sum higher than −1, Supplementary eTable 1 ) (85.9% of men aged <50 vs. 90.6% of men aged ≥50 years). A similar proportion had an estimated intermediate (5–10%) risk (6.4% for men aged <50 and 7.4% for men ≥50, respectively). Estimated low (<5%) recurrence risk was most prevalent in men <50 years (7.7 vs. 2.2%). During anticoagulant treatment, 0.2% of the men aged <50 and 0.8% of the men aged ≥50 suffered from major bleeding. Men aged ≥50 had a higher prevalence of most comorbidities, except for alcohol-related diseases (5.3% among men aged <50 years vs. 4.8% for men aged ≥50).

**Table 1:** Baseline characteristics of 13,932 men with incident VTE

	<50 year, 21% ( *n* = 2,898)	≥50 year, 79% ( *n* = 11,034)
Age, mean years (SD)	40.3 (7.8)	67.7 (10.3)
Pulmonary embolism % ( *n* )	27.3 (792)	37.3 (4,121)
*Major diseases*		
Previous cancer % ( *n* )	0.9 (26)	5.8 (644)
Rheumatic disorder % ( *n* )	2.6 (75)	6.2 (679)
Recent a ischemic heart disease % ( *n* )	0.5 (15)	3.7 (407)
Congestive Heart Failure % ( *n* )	1.0 (29)	4.4 (486)
Chronic obstructive pulmonary disease % ( *n* )	1.8 (53)	8.5 (942)
Diabetes % ( *n* )	2.6 (76)	8.1 (891)
Chronic renal disease % ( *n* )	1.1 (33)	3.1 (344)
Varicose veins % ( *n* )	2.5 (73)	3.6 (400)
Alcohol related diseases % ( *n* )	5.3 (153)	4.8 (527)
Hypertension previous year % ( *n* )	4.9 (141)	29.2 (3,227)
Recent a trauma % ( *n* )	15.4 (446)	6.8 (746)
Recent a major surgery % ( *n* )	11.7 (338)	9.7 (1070)
* AIM-SHA-RP b risk score *		
AIM-SHA-RP b , low risk % ( *n* )	7.7 (223)	2.0 (225)
AIM-SHA-RP b , intermediate risk % ( *n* )	6.4 (185)	7.4 (811)
AIM-SHA-RP b , high risk % ( *n* )	85.9 (2,490)	90.6 (9,998)
*Other characteristics*		
Bleeding during treatment % ( *n* )	0.2 (6)	0.8 (87)
Recent a pneumonia % ( *n* )	4.2 (123)	7.4 (812)
Recent a sepsis % ( *n* )	0.8 (24)	0.9 (101)
Recent a central venous catheter	1.0 (30)	1.0 (105)
Previous gastrointestinal bleeding % ( *n* )	0.6 (16)	1.9 (213)
Statin treatment previous year % ( *n* )	4.1 (118)	23.4 (2,583)
Aspirin treatment previous year % ( *n* )	2.0 (59)	18.6 (2,048)
Inflammatory bowel disease % ( *n* )	2.0 (58)	1.4 (153)
Thrombophilia % ( *n* )	0.9 (25)	0.3 (34)
Obesity % ( *n* )	4.5 (131)	3.9 (429)

Abbreviations: *n* , numbers; SD, standard deviation; VTE, venous thromboembolism.

a Within 90 d.

b AIM-SHA-RP risk score: Men: Age >50 = +1 point, incident PE = +1 point, recent major surgery = −2 points, statin treatment = −1 point, previous heart disease = +1 point, antiplatelet treatment = −1 point; Women: age >60 = +2 points, incident PE = +1 point, recent major surgery: −2 points, chronic renal disease = −1, recent pneumonia or sepsis = −1 point. Score sum: Men/women: low risk (<5% recurrence risk): < − 1/< 0, intermediate risk (5–10% recurrence risk): −1/0–2, high risk (>10% recurrence risk): > − 1/> 2.

**Fig. 2:**
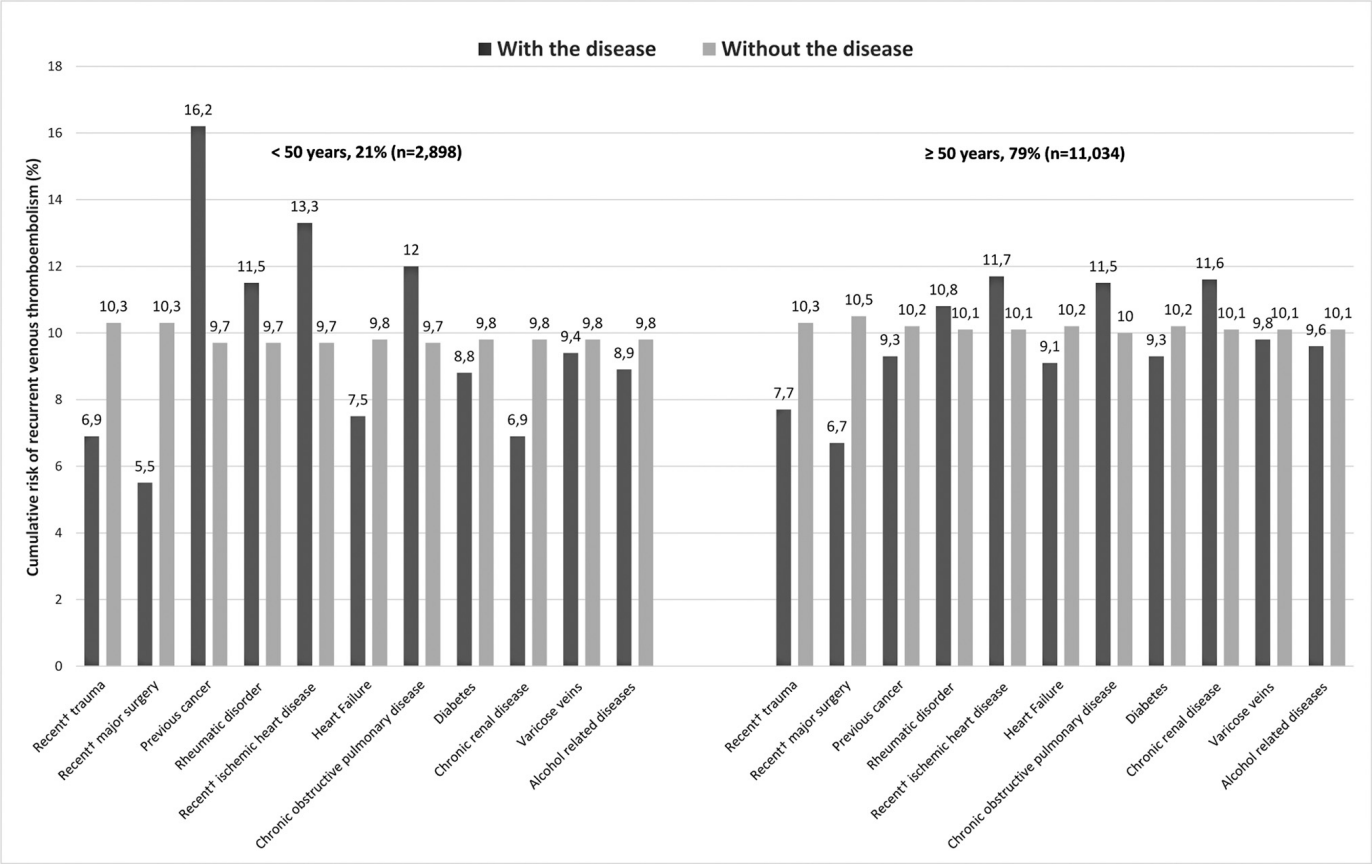
Cumulative venous thromboembolism recurrence risk for men *with* and *without* selected characteristics.

Estimates of cumulative recurrence risk are presented in Table 2 , and recurrence rates are presented in Supplementary eTable 3 . Fig. 2 depicts the 2-year cumulative VTE recurrence risk for men aged <50 and men aged ≥50 years with and without the selected patient characteristics, respectively. For men aged <50 with one of the selected characteristics, recurrence risk varied from 6% (recent major surgery) to 16% (previous cancer) ( Fig. 2 ). For men aged <50 years without the selected patient characteristic, the 2-year recurrence risk was 10% across all subgroups.

**Table 2:** Cumulative recurrence risk at 2 year after anticoagulant treatment discontinuation according to selected patient characteristics, stratified by age

	<50 year, 21% ( *n* = 2,898)		≥50 year, 79% ( *n* = 11,034)	
Characteristic % (95% CI)	With the disease (95% CI)	Without the disease (95% CI)	With the disease (95% CI)	Without the disease (95% CI)
Recent a trauma	6.9 (4.7–9.6)	10.3 (9.1–11.6)	7.7 (5.9–9.9)	10.3 (9.7–10.9)
Recent a major surgery	5.5 (3.4–8.4)	10.3 (9.1–11.6)	6.7 (5.2–8.3)	10.5 (9.9–11.1)
Previous cancer	16.2 (5.1–32.9)	9.7 (8.6–10.8)	9.3 (7.1–11.8)	10.2 (9.6–10.8)
Rheumatic disorder	11.5 (5.3–20.2)	9.7 (8.6–10.9)	10.8 (8.6–13.4)	10.1 (9.5–10.7)
Recent a ischemic heart disease	13.3 (2.2–34.6)	9.7 (8.6–10.9)	11.7 (8.7–15.2)	10.1 (9.5–10.7)
Heart failure	7.5 (1.3–21.3)	9.8 (8.7–10.9)	9.1 (6.7–12.0)	10.2 (9.6–10.8)
COPD	12.0 (4.8–22.7)	9.7 (8.6–10.9)	11.5 (9.5–13.7)	10.0 (9.4–10.6)
Diabetes	8.8 (3.6–17.0)	9.8 (8.7–10.9)	9.3 (7.5–11.4)	10.2 (9.6–10.8)
Chronic renal disease	6.9 (1.2–19.9)	9.8 (8.7–10.9)	11.6 (8.4–15.3)	10.1 (9.5–10.7)
Varicose veins	9.4 (3.8–18.1)	9.8 (8.7–10.9)	9.8 (7.0–13.1)	10.1 (9.5–10.7)
Alcohol-related diseases	8.9 (5.0–14.2)	9.8 (8.7–11.0)	9.6 (7.2–12.4)	10.1 (9.5–10.8)
Hypertension within 1 y	10.8 (6.2–16.9)	9.7 (8.6–10.9)	10.0 (8.9–11.1)	10.2 (9.5–10.9)

Abbreviations: COPD, chronic obstructive pulmonary disease; VTE, venous thromboembolism.

a Within 90 d.

Among men aged ≥50 years with the selected characteristic, recurrence risk ranged from 7% (recent major surgery) to 12% (for ischemic heart disease, chronic obstructive pulmonary disease, and chronic renal disease) ( Fig. 2 ). For men aged ≥50 without such characteristics, the 2-year recurrence risk was 10% for all groups ( Fig. 2 ).

### Supplementary and Sensitivity Analyses

Restricting the analyses to patients with DVT and PE yielded virtually unchanged estimates ( Supplementary eTable 4 ). Of note, male PE patients aged <50 with recent major surgery and recent trauma had the lowest 2-year recurrence risk of 4 and 3%, respectively. All remaining groups had a 2-year recurrence risk of at least 6%. Corresponding risk for DVT patients aged <50 was 6% for patients with recent major surgery vs. 8% for patients with recent trauma.

Extending the initial treatment period to 18 months did not change the results (not shown). Stratifying according to the median age (below/above 63 years) and the 75% percentile for age (below/above 73 years) revealed a 2-year recurrence risk of 4% for patients aged ≥73 with alcohol-related diseases, whereas all remaining subgroups had a recurrence risk of at least 6% ( Supplementary eTable 5 ).

When extending follow-up to 5 years, recurrence risk for men aged <50 years ( Supplementary eFig. 1A ) with one of the selected characteristics ranging from 12% (recent trauma) to 18% (chronic obstructive pulmonary disease). For men aged <50 years without the diseases, the recurrence risk was 18% ( Supplementary eFig. 1B ). For men aged ≥50 with the selected characteristics, recurrence risk ranged from 12% (recent surgery) to 16% (alcohol related disease) ( Supplementary eFig. 2A ). For men aged ≥50 years without the selected characteristics, the 2-year recurrence risk was 19% ( Supplementary eFig. 2B ).

## Discussion

In this large nationwide cohort of men with incident VTE, we observed an overall 2-year recurrence risk of at least 6% after anticoagulation was discontinued. In the main analysis, patients with recent major surgery had the lowest recurrence risk of 6% for men aged <50 and 7% for men aged ≥50. However, in a sensitivity analysis, men with PE aged <50 with recent trauma or surgery had a lower 2-year recurrence risk of 3 and 4%, respectively.

It is well-established that men have a higher risk of recurrent VTE than women. A previous Danish study using a similar VTE cohort as ours investigated VTE recurrence risk for both men and women within 2 years after completed anticoagulant treatment from 2012 through 2017. 20 Among women, 7% had a VTE recurrence risk of <5% 2 years after anticoagulation discontinuation, 73% had a recurrence risk ranging from 5 to 10%, and 20% had a recurrence risk >10%. For men, 3% had a recurrence risk <5%, 7% had intermediate risk of 5 to 10% and 90% had a risk >10%. In clinical practice, the treating physician may put extra focus on the high-risk groups in which the recurrence risk will outweigh the bleeding risk with more certainty. With 90% of the men in the high-risk category compared with only 20% of the women, these results support the concept of more men receiving extended anticoagulation without a scheduled end date. In the 2019 systematic review and meta-analysis on patients with “unprovoked” VTE, the overall 2-year cumulative recurrence risk was 16% (95% confidence interval 13–19%). 7 For men, the 2-year cumulative recurrence risk was 18.3% (95% confidence interval 14.4–22.5%) and for women 13.6% (95% confidence interval 10.1–17.5%), respectively. In a meta-analysis from 2006, the recurrence risk was 50% higher in men compared with women after stopping anticoagulant treatment. 5 In 2008, Baglin et al concluded that male sex was the strongest indicator of recurrence risk (adjusted hazard ratio 2.9 [95% confidence interval [CI] 1.38; 6.01]). 24 Moreuil et al estimated a 7-year recurrence risk of 35% for men and 11% for women. 25 Yet, because there was no difference according to sex in all subgroups, the study concluded that there were no sex-related differences in recurrence risk.

The clinical decision on whether to continue anticoagulant treatment remains complicated. When deciding if a patient should receive indefinite anticoagulation, the expected absolute reduction in major complications and deaths from PE needs to be balanced against the expected increase in major complications and deaths from bleeding. The ESC guidelines have defined a VTE recurrence risk threshold to support the clinical decision of when to safely stop treatment. In the 2019 European Society of Cardiology PE guidelines, only patients with estimated low recurrence risk (<3% per year) are recommended for shorter time-limited treatment, whereas all other patients should be considered for extended treatment without an end date. 8 The ISTH guidelines suggest that it is safe to stop anticoagulation in those whose recurrence risk 1 year after stopping treatment is <5%. 26 The American Society of Haematology does not recommend a specific time for when to discontinue treatment but suggests that DVT and/or PE provoked by a persistent risk factor as well as the majority of patients with unprovoked VTE should receive indefinite duration antithrombotic therapy. 27

The 2019 European Society of Cardiology PE guidelines no longer support the terminology “provoked” and “unprovoked,” as it is “potentially misleading and not helpful for decision-making regarding the duration of anticoagulation.” 8 Instead, a long-term recurrence risk should be estimated according to transient or reversible risk factors. Accordingly, in this study, we estimated recurrence risk according to the major diseases seen in clinic by the treating physician.

European guidelines have initiated a shift in thinking toward extended thromboprophylaxis for patients with an annual risk of recurrence of 3% or higher. However, a study from 2021 described that less than 3% of patients with incident VTE received extended treatment after initial standard treatment, suggesting that this recommendation has not been fully implemented in clinical practice. 28 Also, the counterbalanced risks and consequences of anticoagulant-related major bleeding are considerable. A 2021 systematic review and meta-analysis including 27 studies presented a 5-year cumulative incidence of major bleeding with vitamin K antagonists of 6.3% (95% CI 3.6; 10.0) with a case-fatality rate of 8.3% (95% CI 5.1; 12.2). 29 Finding a recurrence risk of 6% must be placed in a clinical context of patient preferences and bleeding risks. In evidence-based guidelines, it is not recommended to consider male sex as a risk factor when estimating recurrence risk. While perhaps male gender alone should not be an absolute indication, it may be included in the recommendations as a consideration. By analogy, female sex is considered a factor in the CHA _2_ DS _2_ -VASc score used to support treatment decision for atrial fibrillation patients. Finally, if the high VTE recurrence risk for men is confirmed by other studies from diverse populations, future guideline recommendations may place greater emphasis on male sex as a risk factor for recurrent VTE.

Attempts to refine risk prediction for VTE recurrence have been made. 20 30 31 32 33 34 However, most of these prediction models were developed only for patients with “unprovoked,” 30 31 32 33 and the models have been sparsely validated, 35 36 37 38 and none are implemented in the guidelines. We used the AIM-SHA-RP risk score in the current study. 20 This score was originally developed on a cohort of Danish routine care in- and outpatients with completed anticoagulant treatment for incident VTE, 20 and some of these patients overlap with the current study. In the HER DOO2 study, the investigators were unable to identify men with a low recurrence risk. 30 In the remaining models, male sex was a predictor of higher risk of recurrence. To our knowledge, this is the only study trying to refine this overall higher risk described among men.

Some factors may lead to underestimation of the recurrence risk; not including emergency ward diagnoses, and fatal VTE's never registered as VTE events. On the other hand, some VTE ICD-10 codes may not reflect an actual incident event because of inaccurate coding and misclassification leading to overestimation. However, we tried to minimize this risk by only including incident VTE patients initiating anticoagulation within 30 days, ensuring a positive predictive value of 90%. 14 Likewise, some recurrent VTE codes may reflect repeated coding of the incident event and not an actual recurrent VTE. However, by requiring primary recurrent VTE diagnosis to be in combination with relevant imaging examinations, and by starting follow-up after anticoagulant treatment cessation, we ensured a positive predictive value of 82% for the recurrent diagnosis. 21 The trauma and surgery codes, on the other hand, have not been validated for VTE use, potentially introducing some degree of information bias stemming from underlying misclassification. Also, new diseases/conditions occurring during follow-up were not considered in the analysis. Unfortunately, the ICD codes neither differentiated in proximal/distal DVT nor in PE location (central/subsegmental), which is a limitation of our study. Finally, the Danish population may not be representative of other populations with greater variability in race and ethnicity, possibly affecting the external validity of our results.

We followed patients in national registries with prospectively collected data and virtually complete follow-up in a setting with free access to health services, thus largely eliminating selection bias. 12 Of note, the nature of our study was descriptive and therefore confounding was not a concern.

Our study underscores the overall high recurrence risk among men. Male PE patients aged <50 with recent major surgery and recent trauma had a 2-year recurrence risk of 4 and 3%, respectively. However, for all other men, even after taking different risk factors into account, the recurrence risk remained above 6%.

## Notes

**What Is Known on This Topic?**

- Men have a higher risk of recurrent venous thrombosis than do women. However, sex-specific data on prognostic factors for venous thromboembolism recurrence risk are lacking.
- In evidence-based guidelines, male sex has not been considered a risk factor when estimating venous thromboembolism recurrence risk.

**What Does This Paper Add?**

- In this nationwide cohort study, we estimated the cumulative recurrence risks associated with clinical characteristics and comorbidities known or suspected to be associated with the development of VTE recurrence: recent major surgery, recent trauma, history of cancer, rheumatic disorder, recent ischemic heart disease, congestive heart failure, chronic obstructive pulmonary disease, diabetes, chronic renal disease, varicose veins, alcohol-related diseases, arterial hypertension, and age.
- Regardless of age category and disease status, men with venous thromboembolism demonstrated a 2-year recurrence risk after anticoagulation discontinuation of at least 6%.
- All men were above the European guideline threshold of 3% recurrence risk suggesting extended treatment. If the recurrence risk for men is confirmed in diverse populations, future guidelines might put greater emphasize on male sex as a risk factor for recurrent venous thromboembolism.
