# Supplementary material for: Risk of Recurrent Venous Thromboembolism in Selected Subgroups of Men: A Danish Nationwide Cohort Study
Source: TH Open. 2022 Nov 18;6(4):e378–86. doi: 10.1055/a-1949-9404 (PMC9674434; doi:10.1055/a-1949-9404)
Supplement: Supplementary file 1 — Supplementary Material [file 10-1055-a-1949-9404-s22030018.pdf]

Supplementary material

eTable 1: AIM-SHA-RP risk score

eTable 2: ICD 10 codes and ATC codes used in this project

eTable 3: Recurrence rates per 100 person-years according to selected patient characteristics, stratified on age groups.

eTable 4: Cumulative recurrence risk after 2 years follow-up according to selected patient characteristics for DVT and PE patients separately, stratified by age group.

eTable 5: Cumulative recurrence risk at 2 years after anticoagulant treatment discontinuation according to selected patient characteristics, stratified by median age (63 years) and 75% percentile (73 years).

eFigure 1: Cumulative venous thromboembolism recurrence risk for men aged < 50 *with* (A) and *without* (B) selected characteristics at 5 years follow-up.

eFigure 2: Cumulative venous thromboembolism recurrence risk for men aged ≥ 50 *with* (A) and *without* (B) selected characteristics at 5 years follow-up.

eTable 1: AIM-SHA-RP risk score

**AIM-SHA-RP:** Age Incident pulmonary embolism Major surgery – Statin Heart disease Antiplatelet – Renal disease Pneumonia/sepsis			
**Men**		**Women**	
**Variable**	**Points**	**Variable**	**Points**
Age > 50 years	+1	Age > 60 years	+2
Incident pulmonary embolism	+1	Incident pulmonary embolism	+1
Recent Major surgery[a]	-2	Recent Major surgery[a]	-2
Statin treatment[b]	-1	Chronic Renal disease[d]	-1
Previous Heart disease[c]	+1	Recent Pneumonia or sepsis[e]	-1
Antiplatelet treatment[b]	-1		
**Score sum**		**Score sum**	
Low risk: <5%	< -1		<0
Intermediate risk: 5-10%	-1		0-3
High risk: >10%	> -1		> 3

[a]Within 3 months operation with following operation-codes: KA, KB, KD, KF, KG, KH, KJ, KK, KL, KM, KN, KP, KMCA

[b]Treatment within one year before incident venous thromboembolism, ATC-codes: antiplatelet: B01AC04, B01AC06, statins: C10

[c]Within 10 years: stroke, cardiac arrest, ischemic heart disease or congestive heart failure, ICD-10 codes: I60, I61, I62, I63, I64, I46, I20 I21 I22 I23 I24 I25, I50, I110, I130, I132, I420

[d]Within 10 years: chronic kidney disease, unspecified kidney failure, chronic nephritic syndrome, or chronic tubulointerstitial nephritis, ICD-10: N00, N01, N03, N05, I12, I13, I15.0, I15.1, N11, N14, N15, N16, Q61.1-61.4, N18, N19, N26, N27, N07, N08.

[e]Within 3 months hospitalized with sepsis or pneumonia, ICD-10: A40, A41, J12, J13, J14, J15, J16, J17, J18

eTable 2: ICD 10 codes and ATC codes used in this project

	**ICD 10 Codes**	**ATC Codes**
**Venous thromboembolism cohort**		
Deep Venous Thrombosis	I801, I802, I803, I808, I809, I822, I823, I829	
Pulmonary Embolism	I26	
Venous thromboembolism	I26, I801, I802, I803, I808, I809, I828, I829, I822, I823	
**Persistent risk factors (coded up to 10 years before incident VTE)**		
Cancer	C (non-melanoma skin cancer C44 not included)	
Congestive Heart Failure	I50, I110, I130, I132, I420	
Inflammatory bowel disease	K50, K51	
**Other long-term risk factors for VTE (coded up to 10 years before incident VTE)**		
Renal disease	N03, I12, I13, N11, N14, N15, N16, Q61.1-61.4, N18, N19, N26, N27, N07	
Moderate/Severe liver disease	B150, B160, B162, B190, K704, K72, K766, I85	
Alcohol related diseases	E244, E529A, F10, G312, G621, G721, I426, K292, K70, K860, L278A, O354, T51, Z714, Z721	
Chronic obstructive pulmonary disease	J40, J41, J42, J43 J44, J961	

Diabetes	E10, E11, E14	
Hypertension: We identified subjects with hypertension from combination treatment with at least two of the following classes of antihypertensive drugs.		I. Alpha adrenergic blockers (C02A, C02B, C02C)<br>II. Non-loop diuretics (C02DA, C02L, C03A, C03B, C03D, C03E, C03X, C07C, C07D, C08G, C09BA, C09DA, C09XA52)<br>III. Vasodilators (C02DB, C02DD, C02DG, C04, C05)<br>IV. Beta blockers (C07)<br>V. Calcium channel blockers (C07F, C08, C09BB, C09DB)<br>VI. Renin-angiotensin system inhibitors (C09)
Varicose veins	I83	
Rheumatic disorder	M05-14, M46, M47	
AIM-SHA-RP score level		
Bleeding during anticoagulant treatment	K250 K252 K254 K260 K262<br>K264 K270 K272 K274 K280<br>K282 K290 K921 K922 I60 I61 I62	
**Temporary risk factors (coded 3 month before incident VTE)**		

Major surgery	KA, KB, KD, KF, KG, KH, KJ, KK, KL, KM, KN, KP	
Central venous catheter	BMBZ61, BMBZ71, BMBZ51, BMLA01-03	
Fracture/trauma	S00-T14	
**Other short-term risk factors (coded 3 month before incident VTE)**		
Sepsis	A40, A41	
Pneumonia	J12, J13, J14, J15, J16, J17, J18	
Stroke	I60, I61, I62, I63, I64	
Ischemic heart disease	I20 I21 I22 I23 I24 I25	
**Medicine (prescription claims 1 year before incident VTE)**		
Statins		C10
Clopidogrel		B01AC04
Aspirin		B01AC06
Apixaban		B01AF02
Rivaroxaban		B01AF01
Edoxaban		B01AF03
Dabigatran		B01AE07
Warfarin		B01AA03
Phenprocoumon		B01AA04
**Imaging Examinations (used to validate recurrent VTE)**		
Ultrasonography UE	UXUG	

MR venography	UXZ52	
Angiography	UXAG, UXAC10	
Ventilation-perfusion examination	WLHGS	
CT-scan	UXCG, UXCC	
Phlebography	*UXAG05*	
**Exclusion codes**		
Atrial Fibrillation or Flutter	I48	
Mechanical Heart Valve	Z952 Z953 Z954	

eTable 3: Recurrence rates per 100 person-years according to selected patient characteristics, stratified on age groups.

	**< 50 years, 21% (n=2,898)**		**≥ 50 years, 79% (n=11,034)**	
	With the disease (95% CI)	Without the disease (95% CI)	With the disease (95% CI)	Without the disease (95% CI)
Recent† major surgery	2.92 (1.84-4.64)	5.41 (4.77-6.13)	3.52 (2.77-4.47)	5.60 (5.25-5.96)
Recent† trauma	3.60 (2.50-5.18)	5.39 (4.74-6.13)	4.12 (3.16-5.38)	5.48 (5.15-5.84)
History of cancer	8.71 (3.27-23.21)	5.08 (4.50-5.73)	5.11 (3.92-6.65)	5.41 (5.08-5.76)
Rheumatic disorder	5.84 (2.92-11.67)	5.09 (4.50-5.75)	5.92 (4.66-7.50)	5.36 (5.03-5.71)
Recent† ischemic heart disease	7.39 (1.85-29.54)	5.10 (4.52-5.75)	6.48 (4.84-8.68)	5.35 (5.03-5.69)
Heart Failure	3.84 (0.96-15.37)	5.12 (4.54-5.78)	5.40 (5.07-5.74)	5.27 (3.90-7.13)
COPD	6.85 (3.08-15.26)	5.08 (4.50-5.74)	6.50 (5.34-7.89)	5.29 (4.96-5.64)
Diabetes	4.46 (2.00-9.93)	5.13 (4.54-5.79)	5.06 (4.05-6.33)	5.42 (5.09-5.77)
Chronic renal disease	3.21 (0.80-12.82)	5.13 (4.55-5.79)	6.68 (4.86-9.17)	5.35 (5.03-5.69)
Varicose veins	5.12 (4.53-5.78)	4.75 (2.13-10.56)	5.19 (3.74-7.19)	5.40 (5.07-5.74)
Alcohol related diseases	4.86 (2.82-8.37)	5.12 (4.53-5.80)	5.41 (4.08-7.18)	5.39 (5.06-5.74)
Hypertension within previous year	5.48 (3.25-9.25)	5.09 (4.50-5.76)	5.39 (4.82-6.04)	5.39 (5.01-5.79)
VTE= venous thromboembolism, COPD=chronic obstructive pulmonary disease<br>†Within 90 days				

eTable 4: Cumulative recurrence risk after 2 years follow-up according to selected patient characteristics for DVT and PE patients separately, stratified by age group.

	**DVT patients (n=9,019)**				**PE patients (n=4,913)**			
	**< 50 years, 23% (n=2,106)**		**≥ 50 years, 77% (n=6,913)**		**< 50 years, 16% (n=792)**		**≥ 50 years, 84% (n=4,121)**	
	With the disease % (95% CI)	Without the disease % (95% CI)	With the disease % (95% CI)	Without the disease % (95% CI)	With the disease % (95% CI)	Without the disease % (95% CI)	With the disease % (95% CI)	Without the disease % (95% CI)
Recent[†] major surgery	6.1 (3.4-9.8)	10.6 (9.3-12.1)	6.5 (4.7-8.8)	9.9 (9.2-10.7)	4.4 (1.7-9.4)	9.4 (7.2-11.8)	7.7 (5.6-10.5)	10.3 (9.3-11.4)
Recent[†] trauma	8.2 (5.4-11.6)	10.5 (9.1-12.1)	8.3 (5.9-11.2)	9.7 (9.0-10.5)	3.4 (1.1-7.9)	9.6 (7.4-12.1)	7.0 (4.4-10.4)	11.3 (10.2-12.3)
Cancer > 1 year before	19.3 (6.0-38.2)	10.1 (8.8-11.5)	7.9 (5.3-11.2)	9.7 (9.0-10.5)	-	8.7 (6.8-10.9)	11.2 (7.7-15.4)	10.9 (9.9-12.0)
Rheumatic disorder	12.6 (5.1-23.7)	10.1 (8.8-11.5)	10.6 (7.8-14.0)	9.6 (8.8-10.3)	9.0 (1.5-25.0)	8.6 (6.7-10.9)	11.2 (7.6-15.4)	10.9 (9.9-12.0)
Recent[‡] ischemic heart disease	-	10.2 (8.9-11.6)	10.5 (5.9-16.6)	9.6 (8.9-10.4)	22.2 (3.4-51.3)	8.5 (6.6-10.7)	12.3 (8.6-16.6)	10.8 (9.8-11.9)
Heart Failure	-	10.2 (8.9-11.6)	11.7 (7.6-16.8)	9.6 (8.9-10.3)	-	8.6 (6.7-10.8)	7.3 (4.6-10.8)	11.2 (10.2-12.3)
Chronic obstructive pulmonary disease	13.8 (4.3-28.6)	10.1 (8.8-11.5)	12.6 (9.6-16.1)	9.4 (8.7-10.2)	9.4 (1.6-26.0)	8.6 (6.7-10.8)	10.6 (8.0-13.6)	11.0 (9.9-12.1)

Diabetes	9.9 (3.1-21.4)	10.2 (8.9-11.6)	9.3 (6.8-12.2)	9.7 (8.9-10.4)	7.2 (1.3-20.5)	8.7 (6.8-10.9)	9.4 (6.7-12.6)	11.1 (10.1-12.2)
Chronic renal disease	8.7 (1.5-24.3)	10.2 (8.9-11.6)	9.5 (8.8-10.3)	14.1 (9.4-19.6)	..	8.7 (6.8-10.9)	8.5 (4.8-13.6)	11.0 (10.0-12.1)
Varicose veins	9.9 (3.6-20.0)	10.2 (8.9-11.6)	8.6 (5.4-12.6)	9.7 (9.0-10.4)	..	8.7 (6.7-10.9)	12.0 (7.2-18.2)	10.9 (9.9-11.9)
Alcohol related diseases	8.8 (4.1-15.7)	10.2 (8.9-11.7)	9.3 (6.5-12.8)	9.7 (8.9-10.4)	9.1 (3.3-18.5)	8.6 (6.6-10.9)	10.2 (6.3-15.2)	11.0 (10.0-12.0)
Hypertension within previous year	11.0 (5.6-18.4)	10.1 (8.8-11.5)	9.5 (8.2-11.0)	9.7 (8.8-10.5)	10.7 (3.4-22.8)	8.5 (6.6-10.8)	10.5 (8.9-12.3)	11.1 (9.9-12.4)

DVT= deep venous thrombosis, PE= pulmonary embolism, VTE= venous thromboembolism.

†Within 90 days

eTable 5: Cumulative recurrence risk at 2 years after anticoagulant treatment discontinuation according to selected patient characteristics, stratified by median age (63 years) and 75% percentile (73 years).

	**< 63 years, (n=6,623)**		**≥ 63 years, (n=7,309)**		**< 73 years, (n=10,366)**		**≥ 73 years, (n=3,566)**	
Characteristic % (n)	With the disease	Without the disease	With the disease	Without the disease	With the disease	Without the disease	With the disease	Without the disease
Recent† major surgery	5.9 (4.4-7.8)	10.4 (9.6-11.2)	6.9 (5.2-9.0)	10.5 (9.8-11.3)	6.2 (4.8-7.8)	10.6 (10.0-11.3)	7.1 (4.6-10.3)	9.9 (8.8-11.0)
Recent† trauma (any)	7.0 (5.3-9.0)	10.3 (9.5-11.1)	8.2 (5.7-11.3)	10.3 (9.6-11.1)	7.0 (5.5-8.8)	10.5 (9.9-11.2)	9.3 (5.6-14.0)	9.6 (8.6-10.7)
Previous cancer	14.3 (8.4-21.8)	9.8 (9.1-10.6)	8.6 (6.3-11.2)	10.3 (9.6-11.1)	10.2 (0.7-14.0)	10.2 (9.6-10.8)	9.0 (6.2-12.4)	9.7 (8.6-10.8)
Rheumatic disorder	10.6 (7.2-14.6)	9.8 (9.1-10.6)	11.1 (8.3-14.3)	10.1 (9.4-10.9)	11.4 (8.8-14.4)	10.1 (9.5-10.7)	9.8 (6.2-14.2)	9.6 (8.6-10.7)
Recent‡ ischemic heart disease	6.7 (2.7-13.1)	9.9 (9.2-10.7)	13.2 (9.7-17.3)	10.0 (9.3-10.8)	12.2 (8.1-17.2)	10.1 (9.5-10.8)	11.4 (7.5-16.2)	9.5 (8.5-10.6)
Heart Failure	9.2 (4.7-15.5)	9.9 (9.2-10.7)	9.0 (6.4-12.2)	10.2 (9.5-11.0)	10.7 (7.2-15.0)	10.2 (9.6-10.8)	7.4 (4.5-11.1)	9.8 (8.8-10.9)
COPD	8.3 (9.1-10.6)	9.9 (9.2-10.7)	12.5 (10.2-15.1)	9.9 (9.2-10.7)	10.6 (8.0-13.5)	10.2 (9.5-10.8)	12.7 (9.8-16.0)	9.1 (8.1-10.2)

Diabetes	9.4 (6.4-13.1)	9.9 (9.2-10.7)	9.2 (7.1-11.7)	10.3 (9.5-11.0)	8.9 (6.7-11.5)	10.3 (9.6-10.9)	9.9 (7.0-12.4)	9.6 (8.6-10.7)
Chronic renal disease	13.7 (8.0-20.9)	9.8 (9.1-10.6)	10.1 (6.7-14.2)	10.2 (9.5-10.9)	13.3 (9.3-18.7)	10.1 (9.5-10.7)	7.8 (4.3-12.8)	9.7 (8.7-10.8)
Varicose veins	11.2 (7.4-16.9)	9.8 (9.1-10.6)	8.4 (5.2-12.5)	10.2 (9.5-10.9)	10.5 (7.5-14.0)	10.2 (9.6-10.8)	6.7 (2.8-13.2)	9.7 (8.7-10.7)
Alcohol related diseases	10.5 (7.8-13.7)	9.8 (9.1-10.6)	7.7 (4.7-11.5)	10.3 (9.5-11.0)	10.2 (7.9-12.9)	10.2 (9.6-10.8)	4.0 (1.1-10.2)	9.7 (8.8-10.8)
Hypertension within one year	9.9 (7.9-12.1)	9.9 (9.1-10.7)	10.0 (8.9-11.3)	10.3 (9.4-11.2)	10.2 (8.9-11.7)	10.2 (9.5-10.9)	9.7 (8.2-11.4)	9.5 (8.3-10.9)

VTE= venous thromboembolism, COPD=chronic obstructive pulmonary disease

†Within 90 days

eFigure 1: Cumulative venous thromboembolism recurrence risk for men aged < 50 *with* (A) and *without* (B) selected characteristics at 5 years follow-up.

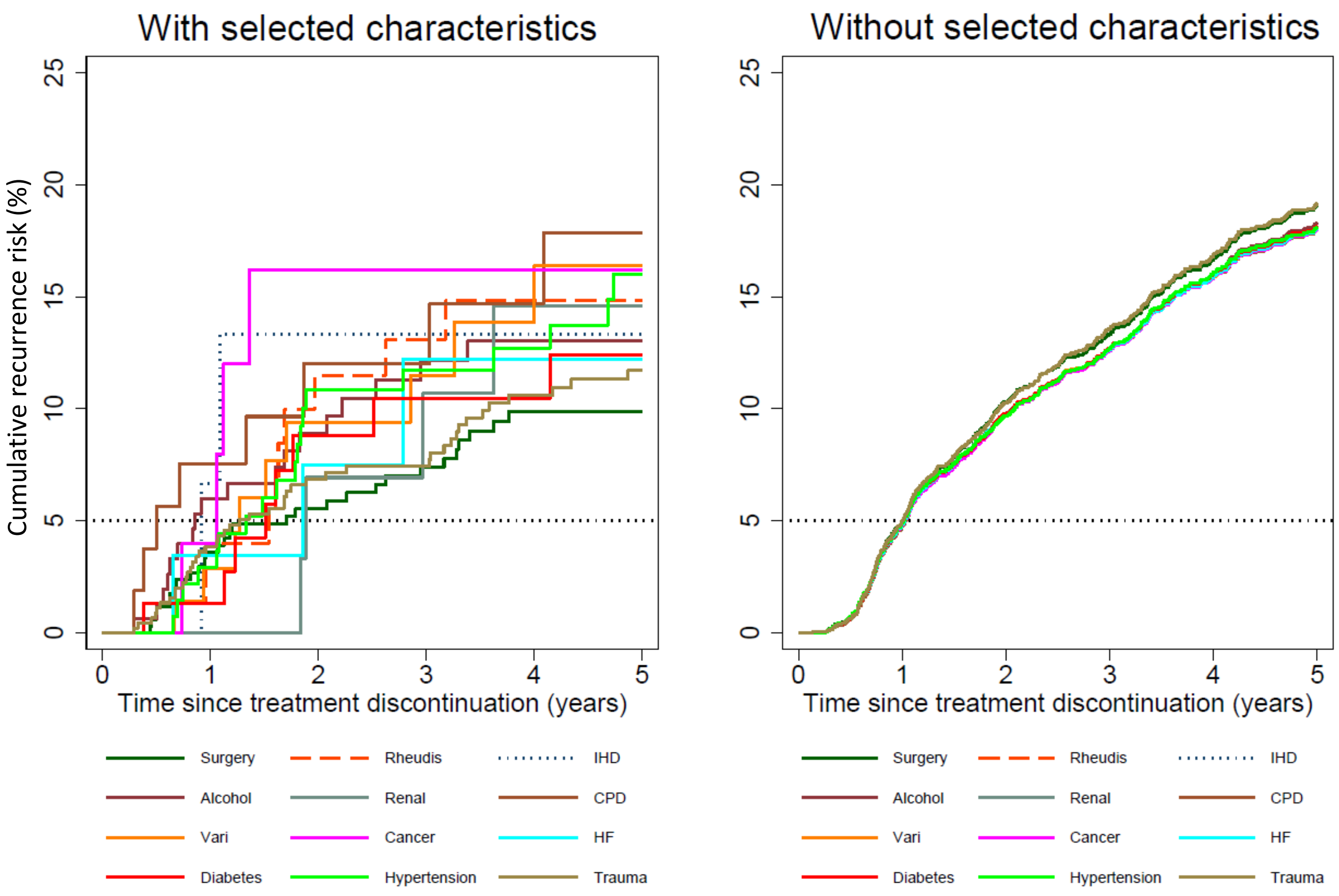

Horizontal dotted line representing 5% indicator for extended treatment according to ISTH.

eFigure 2: Cumulative venous thromboembolism recurrence risk for men aged ≥ 50 *with* (A) and *without* (B) selected characteristics at 5 years follow-up.

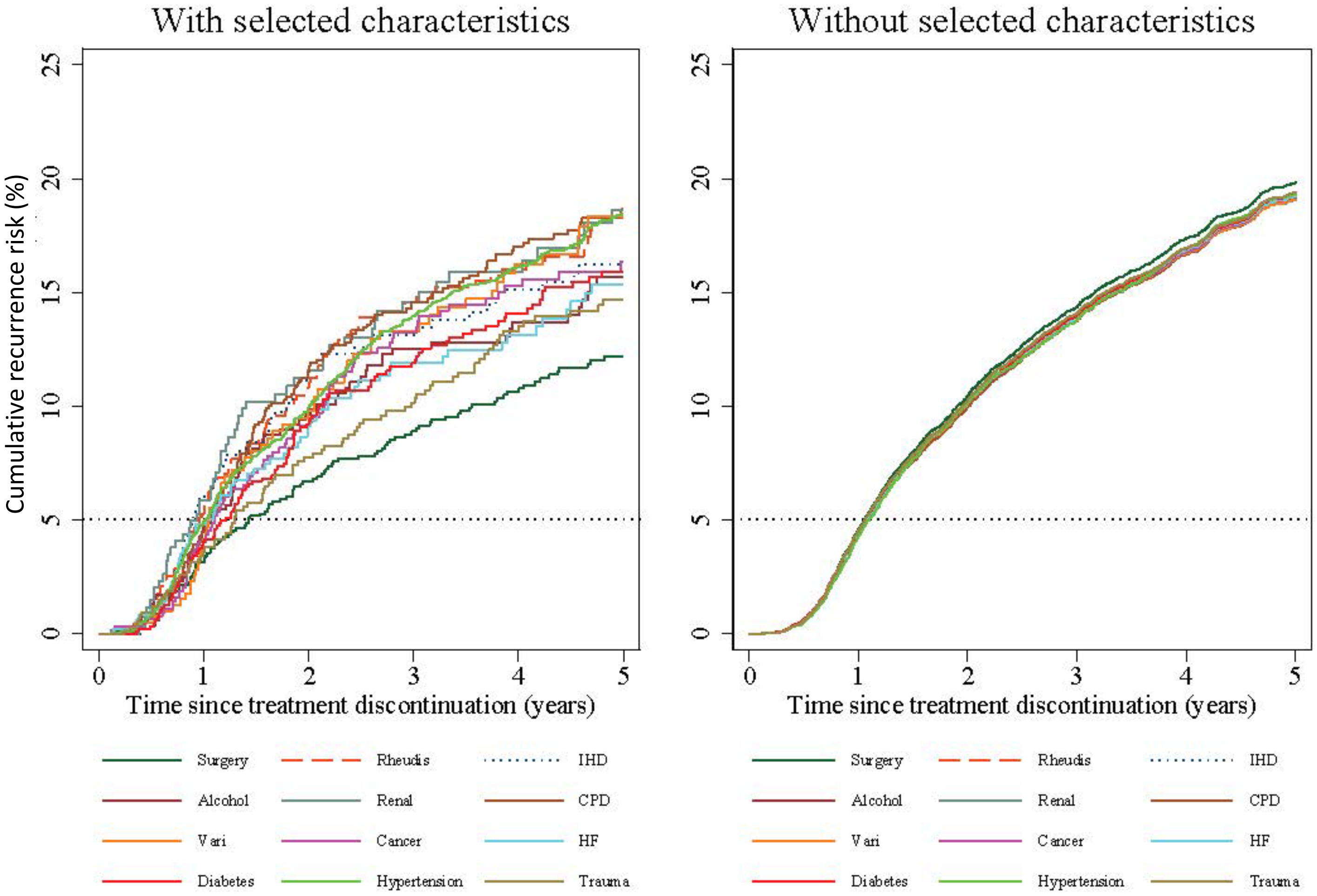

Horizontal dotted line representing 5% indicator for extended treatment according to ISTH.
